# Comprehensive Analysis of Clinical Studies and Regulations of Therapeutic Applications in the United States and Japan

**DOI:** 10.1007/s43441-022-00442-9

**Published:** 2022-09-07

**Authors:** Mao Ono, Kiyotaka Iwasaki

**Affiliations:** 1 Cooperative Major in Advanced Biomedical Sciences, Joint Graduate School of Tokyo Women’s Medical University and Waseda University, Waseda University, 2-2 Wakamatsucho, Shinjuku, Tokyo 162-8480 Japan; 2 Department of Modern Mechanical Engineering, School of Creative Science and Engineering, Waseda University, Tokyo, 169-8555 Japan; 3 Department of Integrative Bioscience and Biomedical Engineering, Graduate School of Advanced Science and Engineering, Waseda University, Tokyo, 162-8480 Japan; 4 Institute for Medical Regulatory Science, Waseda University, Tokyo, 162-8480 Japan

**Keywords:** Digital therapeutics

## Abstract

**Background:**

Digital therapeutics (DTx), the provision of treatment through mobile devices such as smartphones, have attracted great interest as a new medical modality. However, the number of authorized therapeutic applications in the US and Japan is low. Understanding the obstacles in obtaining regulatory authorizations will be the key in promoting timely development of therapeutic applications. Thus, we conducted a comprehensive analysis of the clinical study designs of therapeutic applications authorized in the US and Japan.

**Methods:**

Data on authorized therapeutic applications and the regulations involved were collated from the databases of the Food and Drug Administration (USA), Ministry of Health, Labour and Welfare (Japan), and Pharmaceuticals and Medical Devices Agency (Japan).

**Results:**

Most therapeutic applications authorized targeted neuropsychiatric disorders and used cognitive behavioral therapy (CBT)-based treatments. All the involved clinical trials were randomized-controlled studies. Various types of controls—such as standard care, sham application, digital control, and therapies delivered by healthcare providers—were used. Both subjective and objective indices were acceptable as the primary endpoints. Long-term efficacy was evaluated, and all adverse events were assessed comprehensively. The setting up of controls and the need to study long-term efficacy depend heavily on the applications functionality and the target disease characteristics.

**Conclusions:**

This study reveals the points to be considered in planning clinical studies and regulatory strategies for authorizing therapeutic applications. Therapeutic applications can provide new therapy and have potential to solve unmet clinical needs. Our findings shed a light on efficient development and rapid commercialization of therapeutic applications.

## Introduction

Innovations in medical technology to improve the quality of life, such as those in pharmaceuticals, medical devices, and regenerative medicine, have been remarkable. Recently, digital therapeutics (DTx), that is the provision of treatment through mobile devices such as smartphones, have attracted great interest as a new approach. The Digital Therapeutics Alliance defines DTx as “evidence-based therapeutic interventions that are driven by high-quality software programs to prevent, manage, or treat a medical disorder or disease” [1]. A DTx (intended to treat a disease) is subject to national regulations in the United States and Japan, and its efficacy and safety are evaluated based on clinical study data. Here, we aim to research “therapeutic application” which we define as a DTx used to treat a disease.

The global digital health market was $ 141.8 billion in 2020 and annual growth rate was estimated to be 17.4% for the period 2020 to 2027 [2]. Many DTx startups have partnered with large pharmaceutical companies to develop new DTx [3, 4]. However, the number of authorized DTx including therapeutic applications in the United States and Japan is currently low [5]. How therapeutic applications are evaluated (against conventional therapeutics) through clinical studies has not yet been investigated [6]. Since, therapeutic applications are a new technology and regulations for them are developing and rather unclear, understanding and overcoming obstacles in obtaining market authorization is a key element for their efficient development [5, 7]. Thus, a comprehensive analysis of the currently authorized therapeutic applications will inform developmental research and help advance and optimize future DTx. With a view to promote timely development and regulatory authorization of therapeutic applications, we comprehensively analyzed the authorized therapeutic applications and the targeted disease types, with a focus on their clinical study design to understand the evaluation of efficacy and safety, and the relevant regulations involved.

## Materials and Methods

**Fig. 1:**
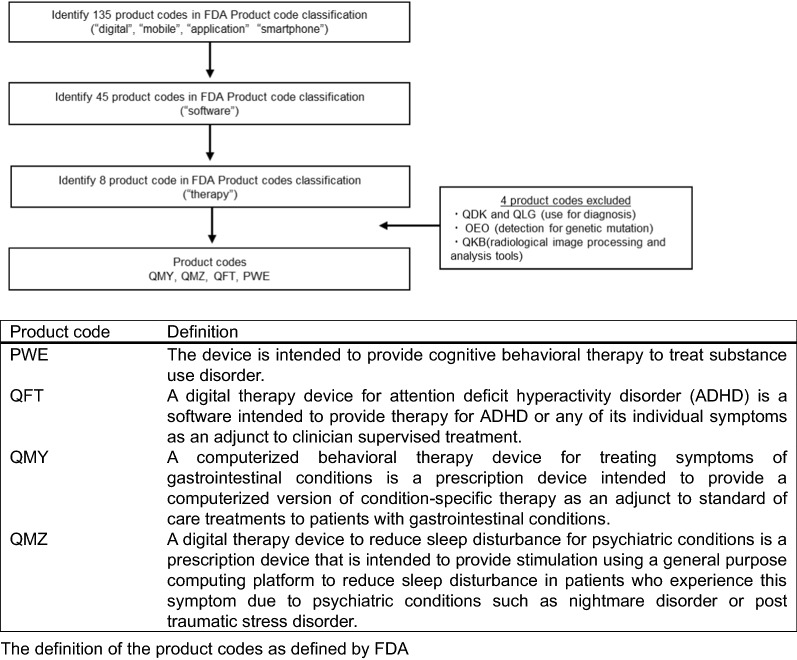
Flow chart to identify the product codes related to therapeutic applications in the United States

We extracted data on authorized therapeutic applications from the Product Code Classification Database [Food and Drug Administration (FDA, United States)] [8]. The product codes were extracted in three steps: (1) first using the terms such as “digital”, “mobile”, “application”, and “smartphone”; (2) then, using the term “software”; and (3) finally, using the term “therapy”. This procedure yielded eight product codes, of which four were excluded for not being therapeutic in functionality (they were either diagnostic, genetic mutation detectors, or radiological image processing tools). Finally, four product codes were included in this study. Data regarding individual medical devices (therapeutic applications) belonging to each of these four extracted product codes were collected (31 July 2021) (Fig. 1).

**Fig. 2:**
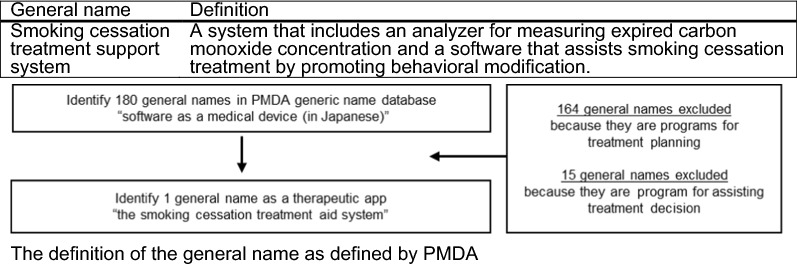
Flow chart to identify the general name related to therapeutic applications in Japan

Data on therapeutic applications approved in Japan were extracted from the General Names Database [Pharmaceuticals and Medical Devices Agency (PMDA), Japan], by searching general names defined under “SaMD (in Japanese)” [9]. One hundred and eighty general names were extracted and classified into three categories: disease diagnosis programs (*n* = 164), disease treatment programs (*n* = 15), and visceral function tests (*n* = 1). General names pertaining to disease diagnosis and disease treatment programs were not considered to be therapeutic application because they fall under treatment planning and decision support programs. Consequently, only one general name was included in this study, and data regarding individual medical devices pertaining to this extracted general name were collected (31 July 2021) (Fig. 2).

We investigated 510(k) summaries, de novo decision summaries, and review reports published by the FDA (USA) and PMDA (Japan) on each product and documents published by the sponsor. Additionally, the product names and ClinicalTrials.gov identifiers were searched in PubMed and Web of Science to obtain information on product summary, clinical study design, efficacy, and safety evaluations.

## Results

### Therapeutic Applications Cleared in the US

Four product codes were extracted from the Food and Drug Administration (FDA) Product Code Classification Database: viz. QMY, QMZ, QFT, and PWE (Fig. 1). Six therapeutic applications—reSET, reSET-O, Somryst, NightWare, EndeavorRx, and Parallel—met these codes (Table 1). The reSET provides cognitive behavioral therapy (CBT) for substance use disorder through texts, videos, etc. [10]. The reSET-O and Somryst deliver CBT for opioid use disorder and chronic insomnia, respectively; reSET-O received 510(k) clearance by establishing substantial equivalence to the predicate device “reSET” [11, 12]. The reSET-O was designated as a Breakthrough Device by the FDA [13]. Somryst was the first product to get clearance under the Digital Health Software Precertification Program, which simplifies the review process for medical device software [13, 14]. EndeavorRx is a video game application for attention-deficit hyperactivity disorder (ADHD) that is indicated to improve attention function [15]. NightWare is a product kit comprising of an application, an iPhone, and an Apple Watch which improves sleep quality by sending vibrations to patients when it senses nightmares; it is aimed at treating sleep disorders mostly in people who suffer from nightmares disorders derived from posttraumatic stress disorder (PTSD) [16, 17]. NightWare was designated as a Breakthrough Device by the FDA [18]. Parallel delivers CBT for irritable bowel syndromes (IBS) through online sessions, and includes personalized interactive tasks [19, 20]. All therapeutic applications cleared in the United States are Class II products. Five out of the six products targeted neuropsychiatric disorders; Parallel (targeting IBS a gastrointestinal disorder) is the only non-neuropsychiatric therapeutic applications. These therapeutic applications are not stand-alone treatment devices or a substitute for medication (Table 1).

**Table 1:** Therapeutic applications authorized in the United States and Japan

Device name	Target patient	Function	Medical specialty	Priority review	Company	Authorization	510(k)/DeNovo
The United States							
reSET	Substance use disorder	Cognitive behavioral therapy modeled on the community reinforcement approach. The content of the therapy lessons is delivered via text, video, animations and graphics.	Neurology	N/A	Pear Therapeutics Inc.	9/14/2017	De Novo
reSET-O	Opioid use disorder	Cognitive behavioral therapy modeled on the community reinforcement approach. The content of the therapy lessons is delivered via text, video, animations and graphics.	Neurology	Breakthrough device	Pear Therapeutics Inc.	12/10/2018	510(k)
Somryst	Chronic insomnia	Cognitive behavioral therapy based on principles of insomnia, sleep restriction, and other proven psychosocial treatment elements. The content of the therapy lessons is delivered via text, video, animations and graphics.	Neurology	Pre-certification program	Pear Therapeutics Inc.	3/23/2020	510(k)
EndeavorRx	Attention deficit hyperactivity disorder	Video game is built on Akili’s proprietary, patented, technology platform and uses adaptive algorithms to deliver stimuli that are designed to engage the patient in a manner that improves their attention function.	Neurology	N/A	Akili Interactive Labs Inc.	6/15/2020	De Novo
NightWare	Nightmare disorder related to PTSD	Smartwatch-based application that senses physiological signals that are consistent with a nightmare utilizing the heart rate sensor, accelerometer, and gyroscope. When the threshold of stress index (device specific indicator) is exceeded, the smartwatch is programmed to provide vibrotactile stimulation on the patient’s wrist to arouse the patient out of the distressed state.	Neurology	Breakthrough device	NightWare, Inc.	11/06/2020	De Novo
Parallel	Irritable bowel syndrome	Cognitive behavioral therapy (CBT) to influence the communication between your brain and gut to improve IBS. The therapy provides guidance based on the personal biological, environmental, and psychological aspects of patients IBS.	Gastroenterology	N/A	Mahana Therapeutics, Inc.	11/25/2020	De Novo
Japan							
The CureApp smoking cessation system	Nicotine dependence	Cognitive behavior therapy accordance with the national guidelines for smoking cessation. The content of the therapy lessons is delivered via messages and educational video, counseling chat sessions and diary.	Respiratory	N/A	CureApp Inc.	8/21/2020	Brand-new

### Therapeutic Applications Approved in Japan

The only general name, the “smoking cessation treatment support system”—extracted from the PMDA General Name Database—supports smoking cessation. It consists of a carbon monoxide (CO)-checker and software as a medical device (SaMD) (Fig. 2). “Smoking cessation treatment support system” corresponds to the CureApp Smoking Cessation System (The CASC system). It provides CBT for nicotine dependent patients through chatbot, animations, etc. [21, 22]. The CASC system is not intended to be used as a stand-alone treatment device and is used in combination with pharmacotherapies. This application is a Class II product and has been approved as a new medical device (a new medical device has a ‘novel structure, usage, indication, or performance’ compared to other medical devices approved in Japan) (Table 1).

### Analyzing the Clinical Study Design Used for the Authorization of Various Therapeutic Applications

All clinical studies submitted for product authorization were randomized (Table 2) [17, 23–28]. Clinical studies evaluating CBT were conducted as an open-label study [23–25, 27, 28]. In contrast, clinical studies for EndeavorRX and NightWare were double blinded [17, 26]. Patients undergoing the usual treatment plan (without use of therapeutic applications, ‘treatment as usual’) were used as control in the reSET and reSET-O studies [23, 24]. For Somryst, a digital health watch without mental intervention was used as control [25, 29]. The clinical trials in Parallel were designed as a three-arm comparative study, and one of the controls was ‘treatment as usual’, and the other was the group that received CBT via healthcare providers [27]. For the CASC system, a sham application (application without content contributing to the treatment) was used as a control. However, since the control group were not provided with CO-checker, they were likely to know that they were the control [21, 22, 28]. EndeavorRx, was studied using a sham application (a digital placebo without therapeutic algorithms) as control [26]. Similarly, a sham application (a digital placebo without treatment interventions) was set as a control in the study of NightWare [17]. Being designated as a Breakthrough Device, NightWare was cleared in the interim analysis [30], resulting in a relatively small number of patients in the study compared to the study of other therapeutic applications [18, 31] (Table 2).

**Table 2:** Clinical trial design of therapeutic applications

	Clinical design	Population	Subject group	Control group
reSET	Open-label, randomized	507 Patients	Web-based reSET and reduced treatment as usual	Treatment as usual
reSET-O	Open-label, randomized	170 Patients	Web-based reSET-O and treatment as usual	Treatment as usual
Somryst	Open-label, randomized	1149 Patients	Somryst	Digital control: Health watch contained health and lifestyle web program without specific mental health or sleep-related content.
EndeavorRx	Double-blind, randomized	348 Patients	EndeavorRx	Sham control: the sham app without effective algorithms.
NightWare	Double-blind, randomized	70 Patients	NightWare	Sham control: the sham app consists of the same components as NightWare, but never intervenes during the night.
Parallel	Open-label, randomized	558 Patients	Web-based parallel with minimal therapist support and treatment as usual	Treatment as usual
				Therapist-delivered cognitive behavioral therapy via telephone and treatment as usual
The CureApp smoking cessation system	Open-label, randomized	584 Patients	The CureApp smoking cessation system	Sham control: a sham app without specific mental intervention and mobile CO checker

Objective indices were set as primary endpoints in the clinical study of reSET, reSET-O, the CASC system, and EndeavorRX, (Table 3) [23, 24, 26, 28]. Efficacy of reSET (for substance use disorder) and reSET-O (for opioid use disorder) were evaluated by assessing abstinence (via screening for drug in urine and urine retention) [23, 24]. Similarly, efficacy of CASC system was assessed by monitoring the abstinence rate (using a CO-checker) [28]. EndeavorRX (for ADHD) efficacy was assessed by monitoring the Attention Performance Index (using the Test of Variables of Attention) [26, 32]. Contrarily, subjective indicators (using Patient-Reported Outcome) were used as primary endpoints to assess the efficacies of Somryst, Parallel, and NightWare [17, 25, 27]. Results of Patient Health Questionnaire-9 were used as the primary endpoint to assess efficacy of Somryst (insomnia treatment) [25, 33, 34]. However, the FDA assessed—Insomnia Severity Index (the secondary endpoint in the submitted clinical studies) [35, 36]—as the important endpoint to evaluate Somryst [12]. NightWare (for nightmare disorder) was assessed using the Pittsburgh Sleep Quality Index [17, 37]. Co-primary endpoints—Irritable Bowel Syndromes-Symptom Severity Score [38] and the Work and Social Adjustment Scale [39]—were used to evaluate Parallel (for IBS) [27].

**Table 3:** Efficacy and safety of therapeutic applications

	Efficacy			Safety
	Efficacy endpoint	Treatment duration	Long-term efficacy after treatment	Safety endpoint
reSET	Abstinence at 9–12 weeks and retention in outpatient therapy	12 Weeks	–	Not prescribed
reSET-O	Longest continuous abstinence, total abstinence, and days in retained in the treatment at 12 weeks	12 Weeks	–	Not prescribe
Somryst	Patient health questionnaire at 6 months	9 Weeks	18 Month	Not prescribe
EndeavorRx	Change in the test of variables of attention–attention performance index at 4 weeks	4 Weeks	1 Month	Not prescribed
NightWare	Pittsburgh Sleep Quality Index Scale at 30 days	30 Days	–	Epworth Sleepiness Scale and Columbia Suicide Severity Rating Scale
Parallel	IBS Symptom Severity Score and Work and Social Adjustment Scale at 12 months	12 Weeks	9 Month	Not prescribed
The CureApp smoking cessation system	Continuous abstinence rate from weeks 9–24	24 Weeks	6 Month	Not prescribed

Long-term efficacy was evaluated for reSET, Somryst, EndeavorRX, Parallel, and the CASC system. The study of reSET (for substance use disorder) did not show significant efficacy at 6-month follow-up after the cessation of the treatment, although the efficacy was confirmed just after the treatment (at week 12) [23]. Efficacy of Somryst (for insomnia) did not diminish even after 18 months of cessation following 9-week treatment [25, 40]. Efficacy of Parallel (for IBS) did not diminish at 9 months follow-up after 12-week treatment [27]. The CASC system (for nicotine dependence) showed efficacy up to 6 months after 24-week treatment [28]. EndeavorRX (for ADHD) was tested to verify the durability of its effects under two conditions—1) 1 month after 4-week initial treatment, and 2) with additional treatment. The efficacy remained significant 1 month after the 4-week initial treatment [26, 41]. At present, the long-term efficacy of reSET-O (for opioid use disorder) and NightWare (for nightmare disorder) has not been evaluated; both of them are designated as Breakthrough Devices.

All adverse events that occurred during the clinical studies were evaluated to address safety issues. Of note, the Epworth Sleepiness Scale [42] and the Columbia-Suicide Severity Rating Scale [43] were set as safety endpoints in the clinical study of NightWare, (Table 3) [16, 17].

### Regulatory Policies in the US

**Fig. 3:**
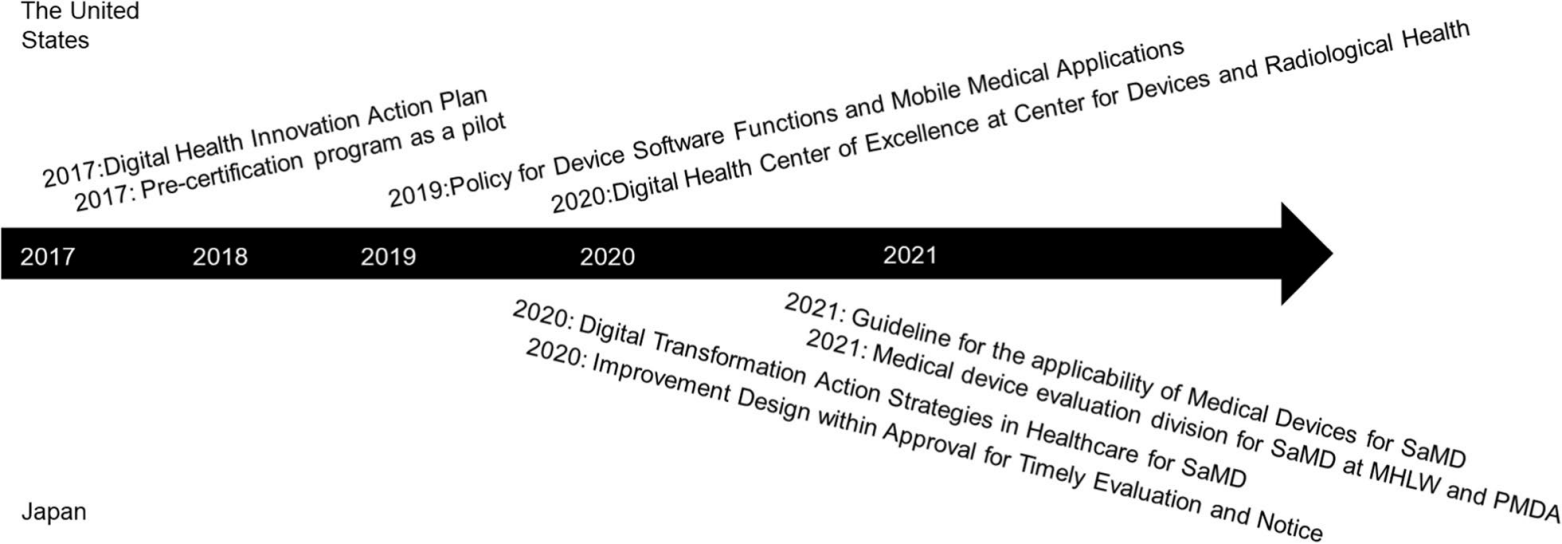
Regulatory policies regarding software as a medical device and/or digital health in the United States and Japan

In the United States, the Digital Health Innovation Action Plan was implemented in 2017 to provide high-quality, safe, timely and effective digital health products for patients [44]. It aims to issue new guidelines to clarify the medical software provision and launch the Precertification Program (as a pilot) to develop a new approach to oversee digital health technology. According to this action plan, “Policy for Device Software Functions and Mobile Medical Applications” was published to elucidate how the FDA intends to apply its regulatory authority to select software applications intended for use on mobile platforms [45]. The Software Precertification Program streamlines the regulatory oversight by pre-certifying sponsors and looks first at the software developer, rather than at individual product [13, 14]. This pilot program will be applied to companies which can demonstrate patient safety, product quality, clinical responsibility, cybersecurity responsibility, and proactive culture. Somryst was developed by Pear Therapeutics (a company covered by this pilot program) and it was the first product cleared under this program [46]. In addition, the Digital Health Center of Excellence was established at the Center for Devices and Radiological Health in 2020 [47]. Thus, the FDA has taken comprehensive initiatives to promote the development of digital health technologies, including DTx (Fig. 3).

### Regulatory Policies in Japan

In Japan, the Digital Transformation Action Strategies in Healthcare (DASH) for Software as Medical Device (SaMD) (also called ‘DASH for SaMD’) was implemented in 2020 [48]. It aims to ensure timely patient access to innovative SaMD via: (1) publishing the basic concept of review; (2) establishing a comprehensive consultation; (3) organizing an optimal review system; and (4) strengthening the regulatory system, for early commercial use. Thus, to accomplish the above four goals, the following have been undertaken—(1) A basic concept of review—a guideline for evaluating SaMD for behavioral therapy—will be issued in 2022 [49]; (2) A new consultation category, for comprehensive SaMD consultation, has been newly established at PMDA (in 2021) with a view to establish a comprehensive consultation process [50]. For instance, previously, the local prefectural office was responsible for deciding the applicability to medical devices. Additionally, the PMDA assesses the efficacy and safety of medical devices, and the Ministry of Health, Labour and Welfare (MHLW) is responsible for healthcare insurance. Consequently, sponsors had to consult with each regulatory authority, separately, depending on the oversight area. However, under the new overhauled consultation/regulatory system, the sponsor applies once for consultation to the PMDA to discuss (the above three topics): applicability to medical devices, the regulatory strategy, and healthcare insurance. This new consultation process saves the sponsor’s resources and contributes to the timely development of products. Furthermore, regulatory authorities may cooperate with each other under this comprehensive consultation process; (3) To organize an optimal review system, a new policy—“Improvement Design within Approval for Timely Evaluation and Notification (named as IDATEN)”—has been introduced for SaMD [51]. IDATEN aims to allow sponsors to conduct continuous improvements. The sponsors have to submit the intended-change plan at the initial submission, and the PMDA reviews it throughout the review period. If the expected change is within the range of the initially submitted plan, a partial change notification is immediately accepted; and (4) Last, to strengthen the regulatory system, the Medical Device Evaluation division for SaMD was established at the PMDA and MHLW [52–54]. Moreover, the “Guideline for the applicability to Medical Devices for SaMD” was published in March 2021 [55] (Fig. 3).

## Discussion

To our knowledge, this is the first study to systematically analyze the currently authorized therapeutic applications in terms of diseases covered, clinical study design, efficacy and safety; we have also studied the regulations involved in the therapeutic applications authorization process in the United States and Japan. Briefly, we found that, all authorized therapeutic applications targeted neuropsychiatric disorders, and most of the treatments provided by therapeutic applications were CBT based. All clinical trials submitted were randomized-controlled studies. The trials were blinded, or open labelled depending on the target disease characteristics and the application functionality. The design of controls varied from study to study, for instance groups on standard care, sham application, digital control, and therapy provided by healthcare professionals were used as controls. Both subjective and objective indices were acceptable as primary endpoints depending on the characteristics of the diseases. Long-term efficacy was evaluated for all therapeutic applications, excepting serious diseases such as nightmare disorder, substance use disorder, and opioid use disorder. All adverse events that occurred during the clinical study were comprehensively evaluated for addressing safety concerns.

### Therapeutic Areas Targeted by Therapeutic Applications Authorized in the US and Japan

Five out (reSET, reSET-O, Somryst, NightWare and EndeavorRx) of the seven therapeutic applications (authorized in the United States and Japan), targeted the neuropsychiatric disorders; Parallel (targets IBS, a gastrointestinal disorder) and the CASC system (targets nicotine dependence, a respiratory disorder). However, notably, IBS and nicotine dependence are associated with mental health or mental conditions [56, 57]. Thus, this study implies that therapeutic applications may help treat the neuropsychiatric diseases which are difficult to treat with current pharmaceuticals, medical devices, and regenerative medicine products.

Of the seven products, five delivered CBT. In current situations, especially in Japan, providing face-to-face CBT sessions is difficult as it requires skilled professionals and is time-consuming. Therefore, CBT is not sufficiently popular in current medical systems [58, 59]. Considering that patients who need CBT can be potentially treated with therapeutic applications, the need for research and development of therapeutic applications focused on diseases only treatable by CBT, is expected to increase.

### The Overview of the Clinical Study Design Used for the Authorization of Various Therapeutic Applications

Evaluation of all the therapeutic applications authorized in the United States and Japan were conducted using randomized-controlled trials. All therapeutic applications other than EndeavorRX (for ADHD) and NightWare (for nightmare disorder) have been assessed using open-label studies. Our results indicate that conducting blinded studies to evaluate efficacy of CBT delivery via therapeutic applications is difficult. Hence, it is acceptable to conduct an open-label study. Rational design of clinical studies—considering the feasibility of a blinded study and the disease characteristics—is important.

For reSET and reSET-O, ‘treatment as usual’ was employed as control against treatments using the applications. Since the target patients had substance use disorder and opioid use disorder, respectively, continuing treatment is critical. Allocating patients to a sham group might lead to decrease in motivation for treatment [21, 22]; thus, standard therapy was selected for the control group. Of note, a digital control was established as a control group for Somryst treating insomnia. A group receiving CBT from healthcare professionals was used as a control instead of a group using a sham application for Parallel treating IBS. Not using a sham control might evoke concerns of assessment biases, however, the biases were eliminated as much as possible using the digital control and the group receiving CBT from healthcare professionals as controls. The CASC system (for nicotine dependence) used a sham application as a control. Nevertheless, it was not a blinded study and patients probably knew which groups they were in because the sham application lacks contents used for treatment. Moreover, the CO-checker was not provided to the control group. For this reason, the regulatory authorities were concerned that using a sham application might decrease treatment motivation and the results might not be accurate. Thus, the regulatory authorities asked the applicant to ascertain if the continuous abstinence rate in the control group is comparable to or higher than that achieved in the standard smoking cessation treatment program implemented in the clinical setting. The sponsor was obliged to explain this matter further as a query response [21, 22]. Use of sham applications in the development of therapeutic applications that provide CBT might not be appropriate due to ethical reasons and technical feasibility; their necessity should be carefully considered. Our study suggests that setting up an optimal control group based on the functions of applications and characteristics of the target disease is indispensable. In addition, the digital control and the group receiving therapy by healthcare professionals could be reasonable control options.

To exclude assessment bias, the efficacy is usually evaluated using endpoints based on objective indices. However, for some diseases, it may be preferable to evaluate efficacy using subjective indices such as Patient-Reported Outcome. For instance, Somryst (for insomnia), NightWare (for nightmare disorder) and Parallel (for IBS), were assessed using subjective scores (validated and widely used in epidemiological research, clinical research, and other studies) as primary endpoints [34, 37–39, 60–63]. Therefore, it is acceptable to use subjective indicators. To demonstrate that biases are eliminated as much as possible, it is noteworthy, to choose validated indices.

The long-term efficacy of reSET gradually decreased at 6-month follow-up after the 12-week treatment. The reSET-O (for opioid use disorder) was cleared without evaluating long-term efficacy. Given that these applications for substance- and opioid- use disorders are used in conjunction with contingency management, continued treatment is meaningful for patients, even though long-term efficacy has not been demonstrated. In addition, the United States has issued a “determination that public health emergency exists” in situations of opioid crisis, thus having a variety of treatment options is of great national importance [64]. In contrast, the CASC system for nicotine dependence has shown efficacy at 1-year follow-up after the start of treatment. Even though substance- and opioid-use disorders, and nicotine dependence are addictions, the seriousness and urgency would vary with type of dependence. Therefore, it is important to consider the risk of the disease itself and its criticality in the country. Long-term efficacy of therapeutic applications for chronic diseases must be evaluated because in chronic diseases therapeutic effects cannot be obtained unless long-term efficacy is shown; short-term approaches do not work. For instance, both Somryst and Parallel have been cleared after long-term efficacy at least 1 year of follow-up was shown. EndeavorRX (for ADHD) showed a continued effect after treatment pause of 1 month. Since physicians are required to determine the retreatment and pausing of treatment depending on the patient’s condition, the measures to evaluate the long-term efficacy of this application could not be standardized. NightWare has been cleared by the interim analysis; the study is still ongoing. Therefore, its long-term efficacy has not yet been assessed. Our findings suggest that demonstrating a continued efficacy at one-year follow-up after the start of treatment by therapeutic applications may be required for chronic diseases. However, the disease characteristics, the usage, function of the applications are important factors when considering the necessity of demonstrating long-term efficacy.

Only the NightWare study set safety endpoints. Its safety was evaluated using The Epworth Sleepiness and the Columbia-Suicide Severity Rating Scales because the treatment could affect daytime sleepiness and suicidal ideation, which are serious symptoms in posttraumatic stress disorder patients with nightmare disorder. In all clinical studies, including NightWare, all adverse events were recorded. These therapeutic applications are Class II products without high-risk. Therefore, to plan adequate risk management in the post-marketing phase, a comprehensive study of the adverse events occurring in clinical trials should be conducted. In addition, safety endpoints need to be established in accordance with disease characteristics dovetailed for specific concerns, as was done in the NightWare safety study.

### Regulatory Policies in the US and Japan

The development of therapeutic applications in the United States has been progressing at a rapid pace compared to that in Japan, as seen from the six therapeutic applications cleared in the United States and only one approved in Japan. The United States has been working on developing regulations in the field of digital health, including therapeutic applications, ahead of Japan. For instance, reSET-O and NightWare were designated as a Breakthrough Device, and Somryst was cleared under the Software Precertification Program.

In Japan, the MHLW has been working to improve the regulations of digital health, including therapeutic applications, since 2020. The evaluation division for SaMD was established in the MHLW and PMDA in April 2021 as one of the accomplishments of DASH for SaMD. In addition, the preparation for issuing guidelines for evaluating SaMD for behavioral therapy is ongoing. Consequently, improvements to strengthen the regulatory system proceeded rapidly. Following the situations in the United States, it is invaluable for regulatory authorities to publish the guidelines and clarify the basic concept in a timely manner, fostering the further development of new modalities such as therapeutic applications.

### Regulatory Policies in Europe and Rest of the World

In Europe, medical devices are certificated as CE Marked under the Medical Device Regulations (MDR). However, no specific legal regulation exists on DTx at a European level [65–67]. On a national level, the new Digital Healthcare Act regulates specific requirements for the use of DTx and provide fast track process for market access under digital health applications (DiGA) in Germany [65, 68]. European Medicines Agency (EMA) has an important role in facilitating the appropriate recognition of DTx and introducing DTx products into the whole European market.

In the United Kingdom, DTx is required to be UK General Data Protection Regulation compliant and must meet the Digital Technology Assessment Criteria requirements [69]. DTx are recognized as Digital Health Technologies under the National Institute for Health and Care Excellence’s (NICE) Evidence for Effectiveness framework [70]. Products can be used under the National Health Service after health economic benefits are demonstrated.

The International Medical Device Regulators Forum (IMDRF) published “Software as a Medical Device: Possible Framework for Risk Categorization and Corresponding Considerations” [71], several international regulatory authorities have introduced guidance to address software qualification. For example, in Australia, the Therapeutic Goods Administration has published “Consultation: Scope of regulated software-based products” which provides a comprehensive overview of software qualification [72]. In Singapore, the Health Sciences Authority provided “Regulatory Guidelines for Software Medical Devices: A Life Cycle Approach” [73] and “Regulatory Guidelines for Telehealth Products” [74]. As for regulatory pathways, the Therapeutic Goods Administration and the Health Sciences Authority support regulation through recognition and reliance models by leveraging the approvals of their reference regulatory agencies; Australia is referring to the EU, Singapore is referring the US, Japan, EU, and Canada [75].

### The Need for International Harmonization of Regulatory Policies

It is important to harmonize regulation in the US, Japan, and Europe to facilitate development of DTx and deliver these products to patients in a timely manner around the world. EndeavorRX has obtained CE marked in Europe and is under a phase-2 clinical study in Japan [76, 77]. Parallel has also obtained CE marking in Europe [78]. CureApp, which developed the CACS system, is looking to expand overseas but the product has not been authorized overseas yet [79]. Understanding what kind of DTx can be approved without additional clinical trials worldwide once they are authorized in the US or Japan would be an important research subject.

### The Global Market in the Neuropsychiatric Area is Expected to Increase Rapidly

According to a global marketplace survey, the expenditure on drugs for substance disorders including opioids and nicotine was USD 13.22 billion in 2016, and is expected to reach USD 27.91 billion by 2025 [80]. The drug expenses for insomnia were USD 4.02 billion in 2020, and is expected to reach USD 4.91 billion by 2026 [81]. The cost of treating IBS was USD 3.19 billion in 2019 and expected to reach USD 5.18 billion in 2026 [82]. The medical cost of diseases which require CBT tends to increase year by year, and the cost of treating them is enormous. Therefore, there is a great expectation for the development of new DTx.

There are some limitations to our study. First, therapeutic applications were identified only by the FDA’s Product Code Classification database and PMDA’s generic name database. Second, we investigated only regulatory measures in the United States and Japan. However, it is worthwhile to analyze the therapeutic applications authorized in the United States and Japan in terms of clinical study design and regulation. In particular, CBT is expected to be investigated and developed further because there are unmet needs which cannot be treated with pharmaceuticals, medical devices, and regenerative medicine products.

## Conclusion

So far, therapeutic applications in the neuropsychiatric area have been commercialized, and most of the treatments provided are CBT-based. In clinical studies of therapeutic applications, it is important to consider how to set a control group. Our study demonstrates that groups on digital control, therapy provided by healthcare professionals, standard therapy, and sham applications, may be used as controls. This study indicates it is essential to rationally set the control group based on the target disease characteristics. For primary endpoint, it is acceptable to not only use objective indices but also subjective indices (such as Patient-Reported Outcome) depending on the target disease characteristics and the objective of clinical trials. It is desirable to confirm that the therapeutic effect is long-term. Given that therapeutic applications are novel noninvasive medical modalities, it is essential to monitor all adverse events comprehensively in clinical studies to clarify the points to be considered in the assessment of safety in post-marketing phase. We believe that the findings in this study will contribute to the efficient research, development, and rapid commercialization of therapeutic applications. therapeutic applications may become an alternative therapy for the patients with diseases that are not adequately treated in the current medical system.
